# The Effects of the Marine-Derived Polysaccharides Laminarin and Chitosan on Aspects of Colonic Health in Pigs Challenged with Dextran Sodium Sulphate

**DOI:** 10.3390/md18050262

**Published:** 2020-05-16

**Authors:** Ruth Rattigan, John V. O’Doherty, Stafford Vigors, Marion T. Ryan, Rocco S. Sebastiano, John J. Callanan, Kevin Thornton, Gaurav Rajauria, Lekha M. Margassery, Alan D. W. Dobson, Niall D. O’Leary, Torres Sweeney

**Affiliations:** 1 School of Agriculture and Food Science, University College Dublin, Belfield, Dublin 4 D04 V1W8, Ireland; ruth.rattigan@ucdconnect.ie (R.R.); john.vodoherty@ucd.ie (J.V.O.); staffordvigors1@ucd.ie (S.V.); gaurav.rajauria@ucd.ie (G.R.); 2 School of Veterinary Medicine, University College Dublin, Belfield, Dublin 4 D04 V1W8, Ireland; marion.ryan@ucd.ie (M.T.R.); Sebastiano.simone@yahoo.it (R.S.S.); kevin.thornton@ucd.ie (K.T.); 3 Ross University School of Veterinary Medicine, St. Kitts, West Indies; SCallanan@rossvet.edu.kn; 4 School of Microbiology, University College Cork, Cork City T12 YN60, Ireland; lekha513@gmail.com (L.M.M.); A.Dobson@ucc.ie (A.D.W.D.); N.OLeary@ucc.ie (N.D.O.); 5 Environmental Research Institute, University College Cork, Cork City T12 YN60, Ireland

**Keywords:** colitis, laminarin, chitosan, dextran sodium sulphate, microbiota, inflammation

## Abstract

This study examined the effects of dietary supplementation with laminarin or chitosan on colonic health in pigs challenged with dextran sodium sulphate (DSS). Weaned pigs were assigned to: (1) a basal diet (n = 22); (2) a basal diet + laminarin (n = 10); and (3) a basal diet + chitosan (n = 10). On d35, the basal group was split, creating four groups: (1) the basal diet (control); (2) the basal diet + DSS; (3) the basal diet + laminarin + DSS; and (4) the basal diet + chitosan + DSS. From d39–42, the pigs were orally challenged with DSS. On d44, colonic tissue/digesta samples were collected. The basal DSS group had reduced growth, higher pathology score and an increased expression of *MMP1*, *IL13* and *IL23* compared with the controls (*p* < 0.05); these parameters were similar between the DSS-challenged groups (*p* > 0.05). In the basal DSS group, the relative abundance of beneficial taxa including *Prevotella* and *Roseburia* were reduced while *Escherichia/Shigella* were increased, compared with the controls (*p* < 0.05). The relative abundance of *Escherichia/Shigella* was reduced and the molar proportions of acetate were increased in the laminarin DSS group compared with the basal DSS group (*p* < 0.01), suggesting that laminarin has potential to prevent pathogen proliferation and enhance the volatile fatty acid profile in the colon in a porcine model of colitis.

## 1. Introduction

Inflammatory bowel diseases (IBD) including ulcerative colitis (UC) and Crohn’s disease are characterised by chronic and recurrent inflammation in the gastrointestinal tract (GIT) [1,2,3]. The aetiology of IBD involves a complex interaction between host genetics, lifestyle/environmental factors and the gut microbiome. The IBD patient loses immunotolerance towards enteric bacteria, resulting in uncontrolled inflammation and epithelial damage [3,4,5]. The composition of the gut microbiota strongly influences the outcome of such an immune interaction, with some microbes promoting immune regulation, while other pathogenic organisms drive inflammation at stressful times in susceptible hosts [6].

Conventional medicines used for the management and treatment of IBDs include aminosalicylates [7], corticosteroids [8] and immunosuppressants [7,8]. These medicines have variable remission responses and many create significant side effects with long-term usage [7,9]. Hence, there is a desire to identify natural bioactives that have anti-microbial and anti-inflammatory activities that could be used to manage IBD without negatively impacting on other aspects of health. Such bioactives would be particularly useful and desirable for patients in remission or with milder forms of disease [10]. To effectively influence the environment of the large intestine, bioactive supplements must be resistant to digestion in the upper gastrointestinal tract. A number of bioactives, such as laminarin and chitosan, have such properties. Laminarin is a seaweed-derived polysaccharide composed of β-(1,3)-linked glucans with β-(1,6)-linked side chains of varying length and distribution [11]. Laminarin is classified as a dietary fibre as the presence of glycosidic bonds make it resistant to hydrolytic enzymes and it is non-digestible in in-vitro studies [12]. The anti-inflammatory potential of laminarin in the intestine of weaned pigs has been clearly identified, with reductions in the expression of pro-inflammatory cytokines [13,14,15,16] and increased mucin expression in the ileum and colon [17,18]. Laminarin also favourably influences microbial populations in the post-weaned pig, as evidenced by a reduction in the abundance of attaching and effacing *Escherichia coli* (AEEC) strains and *Enterobacteriaceae* in the large intestine [13,14,16,19]. Chitosan is another marine-derived polysaccharide that has biological characteristics that make it an interesting candidate. Chitosan is formed by the partial deacetylation of chitin, which is found in the exoskeletons of crustaceans. It comprises copolymers of glucosamine and N-acetylglucosamine [20]. It is resistant to hydrolysis by digestive enzymes [21]. In vitro, chitosan has had inhibitory effects against eight species of pathogenic bacteria [22]. Recently, chitosan reduced the degree of body weight loss, disease activity index scores, histological injury and increased the expression of tight junction proteins in dextran sodium sulphate (DSS)-challenged mice [23]. Oral administration of oligosaccharides of chitosan (chito-oligosaccharide (COS)) to mice in two DSS challenge studies also reduced the clinical signs, reduced mucosal injury, reduced NF-κB activation, supressed TNF-α and IL6 levels [24], prevented colonic shortening and inhibited myeloperoxidase, NF-κB, COX-2, and iNOS activation [25]. However, it is unclear if these beneficial effects observed in mice will be observed in other animal models of intestinal inflammation. 

Therapeutic candidates for the treatment of IBD are often tested using animal models of intestinal inflammation including pig models as the GIT of pigs is both morphologically and physiologically similar to that of humans [26,27,28]. Dextran sodium sulphate, a chemical colitogen which induces epithelial damage, is commonly used to model colitis [29]. Whilst more frequently used in rodents, the number of studies using the DSS model in pigs is increasing. These models often examine the effects of a supplement on the clinical signs (weight loss and incidence of diarrhoea), histological features, the gene expression and protein levels of cytokines, intestinal permeability, myeloperoxidase activity and immunoglobulin concentrations [2,10,26,30,31]. Human colitis and the DSS model share several characteristics including superficial ulceration, mucosal damage, leukocyte infiltration, the production of cytokines and other inflammatory mediators [6]. Thus, the objective of this study was to investigate the effects of the prior consumption of laminarin or chitosan on post- DSS challenge faecal scores, daily gains, histological features, the expression of genes involved in inflammation and the diversity and composition of the colonic microbiota. Considering the previously demonstrated growth enhancing, anti-microbial and anti-inflammatory effects of laminarin and chitosan, it was hypothesised that laminarin or chitosan will remediate colitis-type epithelial damage via the divergent mechanisms including the modification of the enteric microbiota and inflammatory transcriptome in the colon, collectively improving the pathology characteristics of colitis.

## 2. Results

### 2.1. Growth Performance and Faecal Scores

#### 2.1.1. Growth Performance in the Pre-Challenge Period (d0-35)

The effect of dietary treatment on growth performance over the pre-challenge period is presented in Table 1. There was no difference in the initial body weight (BW) and the BW at d35 between any of the dietary groups (*p* > 0.05). Pigs supplemented with laminarin had a lower average daily feed intake (ADFI) compared with the basal group over the 35-day period (*p* < 0.05), however, this did not significantly reduce the average daily gain (ADG) or gain to feed ratio (G:F). 

#### 2.1.2. Growth Performance and Faecal Scores in the DSS Challenge Period (d35-44)

The effects of the treatment on growth performance and faecal scores (FS) for the DSS challenge period are presented in Table 2. The basal DSS group had reduced BW, ADG, ADFI and G:F and an increased FS compared to the controls (*p* < 0.05). The basal DSS group had similar final BW, ADG, ADFI and G:F to the chitosan and laminarin DSS treatment groups (*p* > 0.05). The laminarin and chitosan DSS groups had a higher FS compared with the basal DSS group (*p* < 0.001). 

### 2.2. Colonic Histopathology

**Figure 1:**
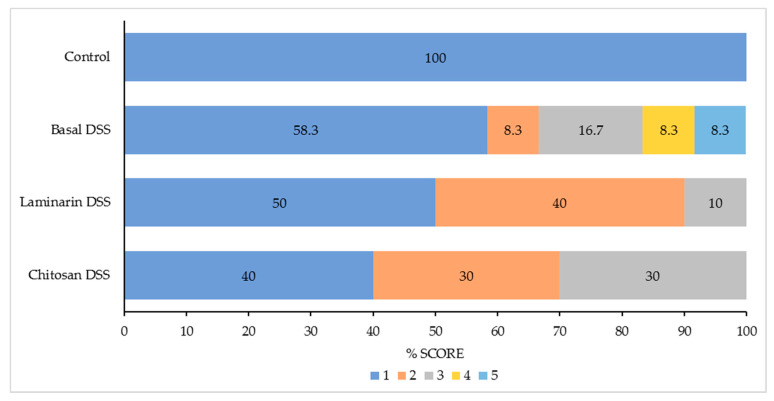
The distribution of the pathology scores between the treatment groups in %. The scoring system was as follows: 1 = normal, non-ulcerating epithelium with a reduced lamina proprial cell infiltration; 2 = normal with a lamina proprial cell infiltration within the normal spectrum; 3 = colonic samples with focal (single) regions of ulceration and 4 = samples with multiple foci of ulceration interspersed between regions of normal colon and 5 = samples with diffuse ulceration. The pathology scores are represented as: score 1 (■), score 2 (■), score 3 (■), score 4 (■) and score 5 (■).

The overall pathology scores and the distribution of the pathology scores within the treatment groups are presented in Table 3 and Figure 1, respectively. All the pigs in the control group had a pathology score of 1 (normal, non-ulcerating colon). In the basal DSS group, 5/12 pigs had a pathology score greater than 1, with scores ranging from 1–5; this profile resulted in the basal DSS group having an overall higher pathology score than the control group (*p* < 0.05). The overall pathology scores were similar between the DSS-challenged groups (*p* > 0.05), however, it must be noted that no animals in the chitosan or laminarin DSS groups had pathology scores greater than 3. 

The goblet/epithelial cell proportions are presented in Table 6. There was a greater proportion of goblet cells and a reduced proportion of epithelial cells in the basal DSS group compared to the control group (*p* < 0.01). The goblet/epithelial cell proportions were similar between the DSS-challenged groups (*p* > 0.05). 

### 2.3. Colonic Gene Expression

The effect of the treatment on gene expression is presented in Table 4. The expression of *MMP1* (*p* < 0.05), *IL13* (*p* < 0.01), and *IL23* (*p* < 0.05) was upregulated in the basal DSS group compared to the control group. There were no differences in the expression of any genes between the basal DSS group and the other DSS-challenged groups. The expressions of *MMP2, MMP9, CLEC7A, TLR4, TJP1, CDH1, IL10, IL17A, IL6, IFNG, TGFB1* and *MUC4* were similar between all the experimental groups.

#### Gene Expression Correlations

**Figure 2:**
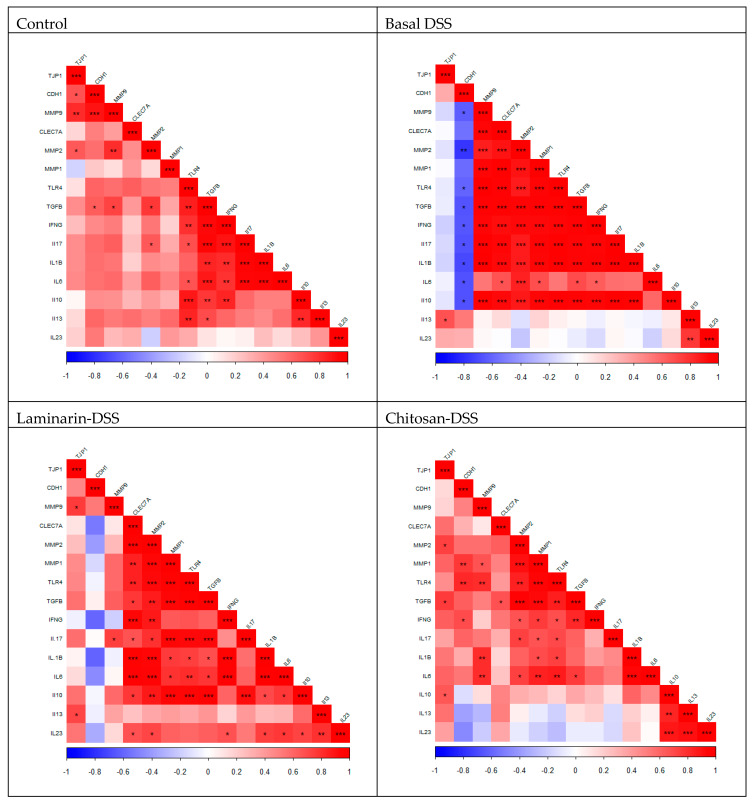
Gene expression correlation matrices for each treatment group. This figure shows the correlation between the measured genes in each treatment group. Positive correlations are indicated in red, while the negative correlations are indicated in blue. The depth of the colour indicates the strength of the positive/negative correlation (0–1). *TJP1*, tight junction protein 1; *CDH1*, cadherin 1; *MMP9*, matrix metalloproteinase 9; *CLEC7A*, C-type lectin domain containing 7A; *MMP2*, matrix metalloproteinase 2; *MMP1*, matrix metalloproteinase 1; *TLR4*, Toll-like receptor 4; *TGFB1*, transforming growth factor beta 1; *IFNG*, interferon gamma; *IL17A*, interleukin 17A; IL1B, interleukin 1B; *IL6*, interleukin 6; *IL10*, interleukin 10; *IL13*, interleukin 13; *IL23A*, interleukin 23 subunit alpha. * *p* < 0.05; ** *p* < 0.01; *** *p* < 0.001.

The gene expression correlation matrices for each of the four treatment groups are presented in the correlogram (Figure 2). There are changes across the groups in the pattern of co-expression indicating a change in the dynamics across the genes being studied. Numerous significant correlations were observed between the genes in all four treatment groups as follows:

Control group: there were 2 clusters of genes which were positively correlated. The first included *TJP1*, *CDH1* and *MMP9* (r = 0.67–0.90; *p* < 0.05). The second comprised *TGFB1*, *IFNG*, *IL6*, *IL1B*, *IL17* (r = 0.79–0.95; *p* < 0.01). Outside of these clusters, *TLR4* positively correlated with *TGFB1*, *IFNG*, *IL17*, *IL6*, *IL10* and *IL13* (r = 0.66–0.89; *p* < 0.05). *TGFB1* positively correlated with *CDH1*, *MMP9*, *MMP2*, *IL10* and *IL13* (r = 0.64–0.77; *p* < 0.05). 

Basal DSS group: *CDH1* negatively correlated with the MMPs; *MMP9* and *MMP2* and with several inflammatory mediators including *TLR4, TGFB1*, *IFNG*, *IL17*, *IL1B, IL6* and *IL10* (r = −0.61–−0.77; *p* < 0.05). There was a large cluster of genes which were strongly and positively correlated including *MMP9, CLEC7A*, *MMP2, MMP1*, *TLR4*, *TGFB1*, *IFNG*, *IL17*, *IL1B* and *IL10* (r = 0.85–1.0; *p* < 0.001). This cluster contained inflammatory cytokines, but as expected it also included all three measured MMPs which are known to be involved in tissue remodelling in IBD. *IL6* positively correlated with *CLEC7A*, *MMP2*, *MMP1*, *TGFB1* and *IFNG* (r = 0.63–0.85; *p* < 0.05) and *IL13* positively correlated with *IL23* (r = 0.77; *p* < 0.01).

Laminarin DSS group: in contrast to the basal DSS group, *CDH1* was not negatively correlated with any gene (*p* > 0.05). There was a cluster of positively correlated genes including *CLEC7A, MMP2, MMP1, TLR4, TGFB1, IL1B*, *IL6* and *IL10* (r = 0.68–0.98; *p* < 0.05). This cluster was similar to the large cluster observed in the basal DSS group, apart from the absence of *IL17* and *MMP9*. *IL23* positively correlated with *IL13*, *IL10*, *CLEC7A*, *MMP2*, *IFNG*, *IL1B*, and *IL6* (r = 0.76–0.80; *p* < 0.05). *IFNG* positively correlated with *CLEC7A*, *MMP2*, *IL1B* and *IL6* (r = 0.88–0.98; *p* < 0.01). *IL17* positively correlated with *MMP9*, *CLEC7A*, *MMP2*, *MMP1*, *TLR4*, *TGFB1*, *IL10* (r = 0.67–0.94; *p* < 0.05). 

Chitosan DSS group: in contrast with the basal DSS group, *CDH1* was positively correlated with *IFNG*, *MMP1* and *TLR4* (r = 0.70–0.82; *p* < 0.05). There were two clusters of positively correlated genes. The first cluster comprised *MMP2, MMP1*, *TLR4*, *TGFB1, IFNG* (r = 0.67–0.97; *p* < 0.05). The second cluster comprised *IL10*, *IL13* and *IL23* (r = 0.84–0.90; *p* < 0.01). Outside of these clusters, *IL17* positively correlated with *MMP2*, *MMP1* and *TLR4* (r = 0.66–0.74; *p* < 0.05). *IL1B* positively correlated with *MMP9*, *MMP1* and *TLR4* (r = 0.70–0.84; *p* < 0.05). *IL6* positively correlated with *MMP9*, *MMP2*, *MMP1*, *TLR4*, *TGFB1* and *IL1B* (r = 0.74–0.95; *p* < 0.05). *TJP1* positively correlated with *MMP2*, *TGFB1* and *IL10* (r = 0.66–0.75; *p* < 0.05). *MMP9* positively correlated with *MMP1* (r = 0.66) and *TLR4* (r = 0.80) (*p* < 0.05).

### 2.4. Volatile Fatty Acids (VFA)

The effect of the treatment on the concentrations of total VFA and molar proportions of VFA is presented in Table 5. There were no differences between treatments on the mmol/gram digesta of total VFA. However, there was a reduction in the molar proportions of acetate (*p* < 0.01) and an increase in propionate (*p* < 0.05), valerate (*p* < 0.01) and branched-chain VFA (*p* < 0.01) in the basal DSS group compared with the control group. There was also a significant increase in the molar proportion of acetate (*p* < 0.05) in the laminarin DSS compared with the basal DSS group. 

### 2.5. Microbiology

#### 2.5.1. Alpha Diversity Analysis

The effect of the treatment on alpha diversity is presented in Table 6. Six of the seven measures of alpha diversity (observed, Chao1, abundance-based coverage estimators (ACE), Shannon, Simpson and Fisher) were reduced in the basal DSS group compared with the control group (*p* < 0.05). One measure of alpha diversity (observed) was increased in the laminarin DSS group compared with the basal DSS group (*p* < 0.05), with no change in diversity detected in the chitosan DSS group. 

#### 2.5.2. Differential Abundance Analysis of Bacterial Taxa in Colonic Digesta

The complete output of the differential abundance analysis (phylum, family, genus, and species levels) in the colonic digesta is presented in the Supplementary Materials (Table S1). The differentially abundant operational taxonomic units (OTUs) at the phylum, family, and genus levels in the colonic digesta are presented in Table 7. The predominant phyla identified were the Firmicutes (53–68%) and Proteobacteria (20–40%), and to a lesser extent the Bacteroidetes (3–7%), and these were found at similar levels in all the experimental groups.

##### Comparison of the Control and the Basal DSS Groups

Phyla: two phyla were differentially abundant; with the Deferribacteres (*p* < 0.001) increased, and the Cyanobacteria reduced (*p* < 0.001) in the basal DSS group compared to the control group. 

OTU: five OTUs within the phylum Firmicutes were differentially abundant between the control and the basal DSS groups. One assigned to the family *Lachnospiraceae* (New.CleanUp.ReferenceOTU8; *p* < 0.05), one assigned to the genus *Phascolarctobacterium* (GQ358246.1.1466; *p* < 0.01), one assigned to the genus *Eubacterium ruminantium* group (EU775472.1.1384), one assigned to the genus *Oribacterium* (EU461618.1.1389; *p* < 0.01), one assigned to the genus *Roseburia* (EU466950.1.1385; *p* < 0.001) and one assigned to the genus *Anaerovibrio* (New.CleanUp.ReferenceOTU1; *p* < 0.001) were all reduced in the basal DSS group.

Five OTUs within the phylum Bacteroidetes were differentially abundant between the control and the basal DSS groups. One assigned to the genus *Bacteroides* (KF842513.1.1417) was increased in the basal DSS group (*p* < 0.001). One assigned to the genus *Rikenellaceae RC9 gut group* (New.CleanUp.ReferenceOTU14; *p* < 0.05), one assigned to the genus *Prevotella 1* (GQ448219.1.1401; *p* < 0.05), one assigned to the genus *Prevotella 7* (EU458732.1.1352; *p* < 0.05) and one assigned to uncultured bacteria within the family *Prevotellaceae* (EF445225.1.1490; *p* < 0.01) were all reduced in the basal DSS group. 

Five OTUs within the phylum Proteobacteria were differentially abundant between the control and the basal DSS groups. One assigned to the family GR-WP33-58 (AF371949.1.1454) and one assigned to the genus *Thalassospira* (New.CleanUp.ReferenceOTU23) were reduced in the basal DSS group (*p* < 0.001), while one assigned to the genus *Escherichia/Shigella* (KF842027.1.1375), and one assigned to the genus *Campylobacter* (AF550648.1.1562) were increased in the basal DSS group (*p* < 0.01). 

One OTU within the phylum Deferribacteres assigned to the genus *Mucispirillum* (AF059190.1.1451) was increased in the basal DSS group (*p* < 0.001). 

One OTU within the phylum Cyanobacteria assigned to uncultured bacteria within the class Gastranaerophilales (JQ184479.1.1344) was reduced in the basal DSS group (*p* < 0.001). 

One OTU within the phylum Synergistes assigned to the family *Synergistaceae* (JQ606918.1.1410) was increased in the basal DSS group (*p* < 0.05).

One OTU within the phylum Tenericutes assigned to uncultured bacterium within the class Mollicutes (DQ805702.1.1378) was reduced in the basal DSS group (*p* < 0.001).

##### Comparison of Basal DSS Group with the Laminarin and Chitosan DSS Groups

Three OTUs within the phylum Proteobacteria were differentially abundant between the basal DSS and the laminarin DSS groups. One assigned to the genus *Succinivibrio* (KF843489.1.1384; *p* < 0.05) was increased, while one assigned to the genus *Succinivibrionaceae* UCG-001 (HQ400282.1.1506; *p* < 0.001) and one assigned to the genus *Escherichia/Shigella* (KF842027.1.1375; *p* < 0.01) were reduced in the laminarin DSS group compared with the basal DSS group. 

One OTU within the phylum Actinobacteria assigned to the genus *Olsenella* (JQ188553.1.1334) was reduced in the laminarin DSS group compared with the basal DSS group (*p* < 0.05). 

One OTU within the phylum Firmicutes assigned to the family *Clostridiales vadin BB60* group (HQ780759.1.1426) was increased in the chitosan DSS group compared with the basal DSS group (*p* < 0.05).

## 3. Discussion

The potential of laminarin or chitosan to protect the colon from the dysbiosis, excessive inflammation and the epithelial injury that typically occurs in an acute DSS challenge model of colitis were investigated. As expected, the acute DSS challenge increased the pathology scores, as well as the markers of inflammation and tissue remodelling in the colon, while it negatively impacted growth performance, faecal scores and colonic microbiota. The supplementation of neither 200 ppm laminarin nor 300 ppm chitosan (MW = 1.526 kDa) was successful in counteracting the effects of the acute DSS challenge in terms of growth performance and inflammation, although none of the pigs in these supplemented groups had a pathology score greater than 3. The pigs supplemented with laminarin had increased molar proportions of acetate and a reduced relative abundance of members of the genus *Escherichia/Shigella,* a genus which is typically increased in IBD patients [32] and associated with severe disease [33]. These changes suggest that laminarin has potential to control pathogen proliferation in an acute experimental pig model of colitis. However, a study of longer duration post-DSS challenge is warranted to ascertain evidence of the resolution of inflammation and tissue remodelling in the colon of the laminarin-supplemented pigs. 

Dysbiosis is commonly reported in studies of IBD and is characterised by an increase in pathogenic bacteria concomitant with a reduction in beneficial bacterial species, although it is not clear whether it is a cause or consequence of inflammation [34]. The microbial profile of the DSS-challenged pigs in this study corresponded with a broad microbial pattern identified in IBD patients, characterised by reduced bacterial diversity, the reduced abundance of bacterial taxa in the phyla Firmicutes and Bacteroidetes and increased Gammaproteobacteria (as reviewed by Zuo and Ng [35]). In this study, basal-fed-DSS-challenged pigs had reduced alpha diversity, characterised by the reduced abundance of taxa within the phyla Firmicutes (*Roseburia, Phascolarctobacterium and Oribacterium*) and Bacteroidetes (*Prevotella* and *Rikenellaceae*) and the increased abundance of genera within the phylum Proteobacteria (*Escherichia/Shigella and Campylobacter)* in colonic digesta. Interestingly, many of the reduced bacterial taxa are associated with VFA production. Previously, *Roseburia* and *Phascolarctobacterium* were reduced in IBD patients [32,36]. The concentrations of total VFAs were unaffected by the DSS challenge, however, there was a reduction in the molar proportions of acetate and increased propionate, valerate and branched-chain fatty acids (BCFA). Intestinal concentrations of BCFA may be used as an indicator of the extent of the microbial amino acid (AA) metabolism [37], as they are produced exclusively through the fermentation of branched-chain AA [38]. Thus, the increase in BCFA suggests that the DSS-challenged pigs had a greater amount of undigested protein reaching the colon. In the laminarin-supplemented pigs, the molar proportions of acetate were increased, suggesting there was more substrate available for bacterial fermentation. The VFAs are essential for the growth and proliferation of colonocytes [39], and while the majority of acetate produced is taken up by the liver and used as an energy source, it may also be used as an energy source for the colonocytes [40]. Thus, this increase in acetate may suggest an improvement in colonic health in laminarin-supplemented pigs. During dysbiosis, facultative anaerobic Proteobacteria including the family *Enterobacteriaceae* expanded at the expense of oxygen-sensitive butyrate producers, disrupting the anaerobic intestinal environment [41,42]. This is consistent with the reduced abundance of *Lachnospiraceae* and *Roseburia* and the increase in *Escherichia/Shigella* in this study. In agreement with the results of this study, a DSS challenge in wild type mice also led to an increase in *Enterobacteriaceae* and *Mucispirillum* and a reduction in *Rikenellaceae* [42]. *E. coli* were previously increased in the mucosa of ulcerative colitis patients and associated with disease activity [43,44]. The *E. coli* strains isolated from IBD patients were frequently adherent and invasive in nature and exhibited more pathogenic potential [45,46]. While many of the *E. coli* present in the human intestine were commensals, during an IBD flare-up these strains may also proliferate and contribute to disease development [47]. Laminarin supplementation caused a reduction in the genus *Escherichia/Shigella*. Laminarin has previously been shown to reduce *Enterobacteriaceae* in newly-weaned and DSS-challenged pigs [48,49] and to reduce *E. coli* levels in weaned pigs [13,50], indicating that laminarin possesses anti-microbial activities against members of the *Enterobacteriaceae* family. As members of the phylum Proteobacteria—particularly *E. coli*—have pro-inflammatory properties and are increased in IBD patients (reviewed by [51]), the ability of laminarin to reduce them suggests that laminarin may have potential to help maintain a healthy microbial profile in IBD patients. 

A number of genes with known relevance to IBD were upregulated in the basal DSS group compared to the untreated controls. These included the cytokines *IL13* and *IL23* and the matrix metalloproteinase *MMP1.* IL13 is a crucial effector cytokine acting on epithelial cell function and initiating apoptosis in UC [52]. Previously, IL13 gene expression was elevated in the colonic mucosa of UC patients [53]. Similarly, serum IL23 levels are increased in UC patients and are proportional to disease severity [54]. IL23 is most highly expressed within the GIT, and it is thought to be involved in the development of a strong immune response against environmental pathogens [55]. MMP1 is upregulated in ulcerated and inflamed areas of the colonic mucosa and its expression levels correlate with disease severity in UC patients [56]. MMPs degrade the extracellular matrix and basement membrane, facilitating cell migration and tissue remodelling [57]. Supplementation with laminarin or chitosan did not reduce the expression levels of the pro-inflammatory genes compared with the basal DSS group. This was surprising as both laminarin and chitosan reduced the expression of a range of pro-inflammatory cytokines in newly weaned pigs—a stressful time characterised by impaired GIT health [13,14,58]. However, the ability of these bioactives to reduce inflammatory responses may vary depending on the degree of challenge to the intestine (as reviewed by Sweeney and O’Doherty [59]). The bioactivity of chitosan varies depending on its degree of deacetylation (DDA) and molecular weight (MW) [60]. A recent study in which chitosan improved the outcome of a DSS challenge in mice [23] used chitosan with a greater DDA (>95% vs. 90.2%) and MW (217kDa vs. 1.526kDa) compared with the chitosan used in the present study. These authors also observed a greater response with 250 vs. 150 ppm chitosan, suggesting that a greater response may have been observed with higher inclusion levels of laminarin and chitosan. The current study was an acute DSS challenge and it is possible that the anti-inflammatory properties may have become evident in a longer-term study once the harsh DSS challenge was removed. As laminarin possesses anti-microbial activities against members of the *Enterobacteriaceae* family which have pro-inflammatory activity, it would be of interest to observe the inflammatory and tissue-remodelling profile of these tissues in a longer-term study. 

While the differences in gene expression levels between the DSS-challenged groups were not significant, it is interesting to note that there were alterations in the pattern of co-expressed genes across the treatment groups suggesting that both laminarin and chitosan were having an influence on the transcriptome. The gene *CDH1* encodes a crucial cell–cell adhesion molecule called E-cadherin that holds epithelial cells together. The expression of *CDH1* was negatively correlated with most cytokines (*IL6*, *IFNG*, *IL10*, *IL17*, and *IL1B*) and MMPs (*MMP2* and *MMP9*) in the basal DSS group. This is in line with other studies, where the expression of E-cadherin was downregulated in biopsy specimens from IBD patients [61,62,63] and is considered a feature of the pathophysiological characteristics of Crohn’s disease, another form of IBD [64]. In the basal DSS group, *MMP9* was positively correlated with several cytokines (*IFNG, IL10, IL17* and *IL1B*) and MMPs (*MMP1* and *MMP2)*. In contrast to the laminarin and chitosan DSS groups, both numbers of correlations (2 and 4, respectively) and strength of the correlations between MMP9 and other genes were reduced. MMP9 has been implicated in the pathogenesis of colitis, with MMP-9^−/−^ mice challenged with DSS having reduced inflammation and mucosal injury, whilst in Caco2-BBE cells, MMP-9 inhibited cell attachment and wound healing [65]. Thus, despite the absence of differences in gene expression levels, the co-expression patterns in the laminarin DSS and chitosan DSS groups differed from the basal DSS group and this may have contributed to the improved distribution of pathology scores within these groups. The signature of expression may be more important in this regard than the effects of individual genes, with the changes to the relationships between these key genes driven by regulatory elements not explored in this study.

No single model of colitis sufficiently represents the complexity of human IBDs. However, the DSS-induced colitis model is frequently used as it is a simple, inexpensive and reproducible model [29], which can be used to study the effects of a dietary supplement on various parameters of colonic health which are affected by IBD. Although the DSS-challenge replicated several of the characteristics observed in human IBD in the current study and several others, there has been a lot of variation between the porcine DSS experiments conducted to date [2,10,26,30,31]. Differences include the ages of the pigs used, the method of DSS administration, the dosage used and the frequency of dosing, the duration of recovery prior to slaughter and the differences in the administration of the compound being tested. The age of the pig used is an important consideration. Many studies have used very young pigs; however, the immune system of neonates is functionally different from that of adults, with the young pig being highly dependent on maternally derived passive immunity [66]. The age at which the pig is weaned and the time between weaning and challenge is also an important consideration as weaning itself is a challenge to the GIT leading to disturbances in gastrointestinal physiology, immunology and microbiology [67]. Thus, these factors should be considered when interpreting the findings of these porcine model studies.

## 4. Materials and Methods 

All experimental procedures described in this work were approved under University College Dublin Animal Research Ethics Committee (AREC-14-14-O’Doherty) and were conducted in accordance with Irish legislation (SI no. 534/2012) and the EU directive 2010/63/EU for animal experimentation. 

### 4.1. Experimental Design and Diets

Forty-two healthy male pigs with an average weight of 9 kg (SD 0.12 kg) were sourced from a commercial pig farm at weaning (~28 days of age) and blocked by weight and litter of origin and housed in pens of two. Initially, each pen was assigned at random to one of three dietary treatments as follows: (1) a basal diet (basal; n = 22); (2) a basal diet + 200 ppm laminarin (Laminarin; n = 10); and (3) a basal diet + 300 ppm chitosan (Chitosan; n = 10). The basal group was assigned 22 pigs for the purpose of a division prior to the DSS challenge, while treatment groups 2 and 3 were assigned 10 pigs each. The concentrations of dietary laminarin (200 ppm) and chitosan (300 ppm) were based on previous studies by Bouwhuis, Sweeney, Mukhopadhya, Thornton, McAlpine and O’Doherty [19] and Xiao, et al. [68], respectively. A laminarin-rich extract was sourced from Bioatlantis Ltd. (Clash Industrial Estate, Tralee, Co. Kerry, Ireland). The laminarin-rich extract was obtained from *Laminaria* spp. using hydrothermal assisted extraction by applying optimized extraction conditions as described previously [69]. The crude extract was partially purified in order to enhance the polysaccharide content and to remove or reduce proteins, polyphenols, and excess amounts of mannitol and alginate, by mixing the crude extract with pure ethanol (to remove polyphenols) followed by water (to remove protein) and calcium chloride (to remove alginates). The extract contained 643 g laminarin/kg, 177 g fucoidan /kg, 60 g/kg ash, 40 g/kg mannitol and 80 g alginate/kg. The laminarin-rich extract was included in sufficient quantity to achieve a concentration of 200 ppm in the relevant treatment. The chitosan was sourced from A&Z Food Additives Co. Ltd. (Hangzhou, China) and had a degree of deacetylation of 90.2% and a molecular weight of 1.526 kDa. The basal diet was the same for all the treatments and was formulated to contain 14.5 MJ/kg of digestible energy, 210 g/kg crude protein and a lysine content of 14.5 g/kg. All amino acid requirements were met relative to lysine [70]. The ingredient composition and chemical analysis of the dietary treatments are presented in Table 8. 

### 4.2. Housing and Animal Management 

The pigs were housed in pairs in fully slated pens (1.7 m × 1.2 m). They were weighed at the beginning of the experiment (d0; day of weaning) and on d35 prior to the DSS challenge and on d44 at the end of the experiment. The ambient environmental temperature within the house was thermostatically controlled at 30 °C for the first 7 days and then reduced by 2 °C per week for the remainder of the experiment. The humidity was maintained at 65%. Feed in the meal form and water were available ad libitum from four-space feeders and nipple drinkers. 

### 4.3. Induction of Experimental Colitis, Clinical Assessment of Colitis and Sample Collection

On d35 the basal group was spilt into two groups for the purpose of the DSS challenge, creating four dietary treatment groups for the remainder of the experiment: (T1) the basal unchallenged group (Control; n = 10), (T2) the basal diet + DSS group (Basal DSS; n = 12), (T3) the basal diet + 200 ppm laminarin + DSS group (Laminarin DSS; n = 10) and (T4) the basal diet + 300 ppm chitosan + DSS group (Chitosan DSS; n = 10). From day 39 to 42, all pigs in the DSS treatment groups were administered DSS solution (0.75 g/kg body weight; molecular weight 47.9 kDa; TdB Consultancy AB, Uppsala, Sweden) via a single daily oral dose, to induce epithelial erosion and mucosal inflammation [10]. The dosage of DSS administered was based on previous work by O’Shea, O’Doherty, Callanan, Doyle, Thornton and Sweeney [48]. Pigs in the control group were administered the same volume of autoclaved water without DSS. Throughout the challenge, faecal scores were recorded daily in individual pens by the same operator, for signs of diarrhoea using the following scale: 1 = hard, firm faeces, 2 = slightly soft faeces, 3 = soft, partially formed faeces, 4 = loose, semi-liquid faeces, and 5 = watery, mucous like faeces [72]. Following the four-day DSS challenge and one-day recovery, all pigs were humanely euthanised by lethal injection with pentobarbitone sodium (Euthanal Solution, 200 mg/mL; Merial Animal Ltd, Essex, UK) at a rate of 0.71 mL/kg body weight. The euthanasia was performed by a trained individual in a room separate from other pigs. The entire digestive tract was immediately removed. The sections from the proximal colon (second loop from the caeco–colic junction) and the distal colon (third loop from the rectum) were excised and fixed in 10% formalin. The digesta from the colon was collected into sterile containers (Sarstedt, Wexford, Ireland) ensuring inter-sample cleaning and the sterilisation of equipment and work surfaces. The samples were snap frozen on dry ice and transferred to −80 °C storage freezers for subsequent microbial and volatile fatty acid (VFA) analyses. In addition, colonic tissue was collected for the gene expression analysis of cytokines, mucins, matrix metalloproteinases (MMPs) and tight junction proteins using QPCR. Sections of colonic tissue (1 cm^2^) were excised, emptied by dissecting along the mesentery and rinsed with sterile PBS (Oxoid, Hampshire, UK). The tissue sections were stripped of the overlying smooth muscle before storage in RNAlater^®^ solution (5 mL) (Applied Biosystems, Foster City, CA, USA) overnight at 4 °C degrees. The RNAlater^®^ was removed prior to storage at −80 °C. 

### 4.4. Feed Analysis

The feed samples were milled through a 1 mm screen (Christy and Norris hammer mill, Ipswich, UK). The dry matter (DM) of the feed was determined after drying overnight at 104 °C. The crude ash content was determined after ignition of a known weight of concentrate in a muffle furnace (Nabertherm, Bremen, Germany) at 550 °C for 6 h. The crude protein (CP) content was determined as Kjeldahl N × 6.25 using the LECO FP 528 instrument. The neutral detergent fibre (NDF) content was determined according to Van Soest [73]. 

### 4.5. Colon Histopathology

For the histopathological assessment, 5 μm sections of colonic tissue were sectioned and stained with haematoxylin and eosin. The stained sections were examined in a blinded fashion by a board-certified veterinary pathologist to evaluate the extent of ulceration as defined by the loss of colonic epithelial cells and an associated variation in the concentrations of lamina proprial cells [74,75]. A histological scoring scheme previously described by O’Shea, O’Doherty, Callanan, Doyle, Thornton and Sweeney [48] was used to categorise the extent of ulceration as follows: 1 = normal, non-ulcerating epithelium with a reduced lamina proprial cell infiltration; 2 = normal with a lamina proprial cell infiltration within the normal spectrum; 3 = colonic samples with focal (single) regions of ulceration and 4 = samples with multiple foci of ulceration interspersed between regions of normal colon and 5 = samples with diffuse ulceration. Samples with scores of 4 and 5 experienced the obliteration of crypt architecture and evidence of acute inflammation with prominent neutrophil margination within blood vessels in the lamina propria. For the enumeration of goblet cells, the colon tissue samples were stained with alcian blue/neutral red and sectioned at 5 μm. The goblet cells were counted as a percentage area of the mucosa in the field of view (4 × objective) using a light microscope fitted with an image analyser (Image-Pro Plus; Media Cybernetics) accounting for the unstained background. The measurements were taken from 15 fields of view per sample. 

### 4.6. Gene Expression Analysis

#### 4.6.1. RNA Extraction and cDNA Synthesis

The total RNA was initially extracted from the colonic tissue using the TRIreagent (Sigma-Aldrich, St. Louis, MO, USA) according to the manufacturer’s instructions. The crude RNA extract was further purified using the GenElute Mammalian Total RNA Miniprep kit (Sigma-Aldrich) which incorporated a DNase step using an on-column DNase 1 Digestion set (Sigma-Aldrich). The total RNA was quantified using a Nanodrop-ND1000 spectrophotometer (Thermo Scientific) and the purity was assessed by determining the ratio of the absorbance at 260 nm and 280 nm. The RNA integrity was assessed using an Agilent 2100 Bioanalyzer with an RNA 6000 Nano LabChip kit (Agilent Technologies, Santa Clara, CA, USA). All samples had a 260:280 ratio > 2.0 and an RNA integrity number (RIN) > 8.0. The total RNA (1 μg) was reverse transcribed using a High Capacity cDNA Reverse Transcription Kit (Applied Biosystems), using oligo (dT) primers in a final reaction volume of 20 μL, according to the manufacturer’s instructions. The cDNA was adjusted to a volume of 120 μL with nuclease-free water. 

#### 4.6.2. Quantitative Real-Time PCR (QPCR)

The mRNA expression profiles of the selected candidate genes were evaluated in duplicate using QPCR on the ABI Prism 7500 Fast Sequence Detection System (Applied Biosystems). Oligonucleotide Primers were designed using the Primer Express Software, version 2.0 (Applied Biosystems) and synthesised by MWG Biotech (Ebersberg, Germany). All the primers for the selected genes, namely interleukin 1B (*IL1B*), interleukin 6 (*IL6*), interleukin 17A (*IL17A*), interleukin 23 subunit alpha (*IL23A*), interleukin 10 (*IL10*), interleukin 13 (*IL13)*, interferon gamma (*IFNG*), transforming growth factor beta 1 (*TGFB1*), tight junction protein 1 (*TJP1*), cadherin-1 (*CDH1*), mucin 4 (*MUC4*), matrix metalloproteinases 1, 2 and 9 (*MMP1, MMP2, MMP9*), Toll-like receptor 4 (*TLR4*) and C-type lectin domain containing 7A (*CLEC7A*), are presented in Table 9. PCR amplification was carried out in a reaction volume of 20 μL, containing 10 μL of Fast SyBr PCR MasterMix (Applied Biosystems), 1.2 μL primer mix (300 mM), 3.8 μL of nuclease-free water and 5 μL of cDNA. The thermal cycling conditions were as follows: 95 °C for 10 min for one cycle, followed by 95 °C for 15 s and 60 °C for 1 min for 40 cycles. All reactions were run in duplicate, minus-RT and no template controls were included, and the dissociation analysis of the PCR products was performed to confirm specificity. The reference genes β−2-microglobulin (*B2M*), glyceraldehyde-3-phosphate dehydrogenase (*GAPDH*), β-actin (*ACTB*), hydroxymethylbilane synthase (*HMBS*), peptidylprolyl isomerase (*PPIA*) and tyrosine 3-monoxygenase/tryptophan 5-monooxygenase activation protein ξ polypeptide (*YWHAZ*) were evaluated for stability using the geNORM function within qBASEPLUS (Biogazelle, Gent, Belgium). The reference genes *PPIA* and *ACTB* were selected as the most stable gene combination and were used for subsequent normalisation. Relative quantities for each target gene were normalised using the normalisation factor calculated from the geometric mean of the most stable reference genes by the geNORM algorithm within qbasePLUS algorithm (Biogazelle). 

### 4.7. Volatile Fatty Acid (VFA) Analysis

The VFA concentrations in the colonic digesta were determined using gas liquid chromatography according to the method described by Pierce, et al. [76]. A 1 g sample was diluted with distilled water (2.5× weight of sample) and centrifuged at 1400× *g* for 10 min using a Sorvall GLC-2B laboratory centrifuge (DuPont, Wilmington, DE, USA). Then, 1 mL of the supernatant and 1 mL of the internal standard (0.05% 3-methyl-n-valeric acid in 0.15 M oxalic acid dihydrate) were mixed with 3 mL of distilled water. The mixture was centrifuged at 500× *g* for 10 min and the supernatant was filtered through a 0.45 PTFE (polytetrafluoroethylene) syringe filter into a chromatographic sample vial. A volume of 1 μL was injected into a Varian 3800 GC (Ontario, Canada) equipped with an EC^TM^ 1000 Grace column (15 m × 0.53 mm I.D) with 1.20 μm film thickness. The temperature programme set was 75 °C–95 °C increasing by 3 °C/min and 95 °C–200 °C increasing by 20 °C/min, which was held for 0.50 min. The detector and injector temperature were 280 °C and 240 °C, respectively, and the total analysis time was 12.42 min.

### 4.8. Microbial Analysis

#### 4.8.1. DNA Isolation and Quantification

Microbial genomic DNA was extracted from 180 mg of colonic digesta samples using the QIAamp DNA Stool Kit in accordance with the manufacturer’s instructions (Qiagen, West Sussex, UK). The DNA purity/integrity was determined via 1% agarose gel electrophoresis (Bio-rad Laboratories Ltd., Watford, Hertfordshire, UK), ethidium bromide staining (Sigma-Aldrich) and UV visualisation (LumiBIS, DNR bioimaging systems, Israel). The quantity of DNA was assessed using a Nanodrop spectrophotometer (NanoDrop ND-1000; Thermo Scientific, Wilmington, DE, USA). The samples were stored at −20 °C.

#### 4.8.2. PCR Amplicon Library Preparation

The regions within the extracted DNA were amplified using polymerase chain reaction (PCR) with primers targeting the V4-V6 hypervariable regions of the 16S rRNA gene, described by the Ribosomal Database project at Michigan State University [77]. The forward primer contained the adapter (fusion primer) sequence (5’-CCATCTCATCCCTGCGTGTCTCCGACTCAG-3’) followed by the six base pair tags/unique barcode sequences listed in Table 10 and the degenerate primer region (5’-AYTGGGYDTAAAGNG-3’). The reverse primer consisted of a common fusion primer (5’-CCTATCCCCTGTGTGCCTTGGCAGTCTCAG-3’) followed by one of four different degenerate primer sequences: PB1 (5’-TACNVGGGTATCTAATCC-3’), PB2 (5-TACCRGGGTHTCTAATCC-3’), PB3 (5’-TACCAGAGTATCTAATTC-3’) and PB4 (5’-CTACDSRGGTMTCTAATC-3’), respectively. The PCR was performed in a total volume of 30 µl which contained sterile water, 1× buffer, 2 mM dNTPs (deoxyribonucleotide triphosphate), 10 ng µl^−1^ template DNA, 0.75 U Taq DNA polymerase (Fermentas, Lithuania) and 10 µM final concentrations of each of the primers. The amplicon (289 bp) was visualized using electrophoresis on a 1.2% agarose gel, with Sybr-Safe (Thermofisher, Waltham, MA) staining and then visualised under UV light. The thermocycling conditions were as follows: initial denaturation (94 °C for 5 min) was followed by 30 cycles of denaturation (94 °C for 30 s), primer annealing (50 °C for 30 s) and primer extension (72 °C for 2 min), with a final primer extension step (72 °C for 10 min). The PCR products were purified using the QIAquick PCR purification kit (Qiagen, Hilden, Germany) according to the manufacturer’s instructions. The purified PCR products were quantified and assessed for purity using a Nanodrop spectrophotometer. The samples were pooled to achieve equimolar concentrations (~1 × 10^11^ copies µL^−1^). The pooled libraries were then supplied to Macrogen Inc., (Seoul, Republic of Korea) for emulsion PCR and pyrosequencing on a GS (genome sequencer) FLX Titanium platform (454 Life Sciences).

#### 4.8.3. Bioinformatic Analyses of Pyrosequencing Reads

The raw reads were initially demultiplexed and quality filtered using the Quantitative Insights into Microbial Ecology (QIIME) package [78]. Errors were corrected using the default program Denoiser [79], while chimeras were identified and removed with the use of UCHIME [80]. OTUs were assigned at 97% sequence similarity based on the SILVA reference database [81]. Representative sequences from each OTU were then aligned to the reference sequences via the QIIME-integrated PyNAST application [82]. The normalized OTU table combined with the phenotype metadata and phylogenetic tree comprised the data matrix. This matrix was then inputted into the phyloseq package within R (http://www.r-project.org; version 3.5.0). The dynamics of richness and diversity in the piglet’s microbiota was computed with the observed, Chao1, ACE, Simpson, Shannon, InvSimpson and Fisher indices. Differential abundance testing was performed using the phyloseq to deseq2 function within R [83,84]. Results are presented using Benjamini–Hochberg (BH) adjusted *p*-values. 

### 4.9. Statistical Analysis

The resulting data were assessed for normality using the univariate procedure of Statistical Analysis Software (SAS). The performance, FS, cytokine, histology data and VFA results were analysed using the PROC MIXED procedure of SAS (Littell et al., 1996). The model used included pen and animal within pen as random effects. The fixed effect was treatment. The least square means were compared between the experimental groups using pre-planned contrast statements. Pre-planned comparisons between the experimental groups were as follows: 1, Control vs. Basal DSS; 2, Basal DSS vs. Laminarin DSS; 3, Basal DSS vs. Chitosan DSS. The probability level that denoted significance was *p* < 0.05. The data was presented in tables as least square means with their standard errors. The colonic histopathology scores were analysed using the PROC Genmod procedure of SAS. Correlograms were created using the package ‘Corrplot’ within R. The corrplot package created a graphical display of a correlation matrix [85,86]. The function, ‘cor.mtest’ produced *p*-values and confidence intervals for each pair of input features, while the argument ‘number’ output the correlation coefficient (colour-coded in the figures).

## 5. Conclusions

In conclusion, the DSS challenge demonstrated effects similar to those observed in IBD patients, including reduced feed intake, weight gain and faecal consistency, increased expression of pro-inflammatory cytokines and matrix metalloproteinases, reduced bacterial diversity, reduced relative abundance of taxa belonging to the Bacteroidetes and Firmicutes, and increased *Escherichia/Shigella*. Whilst laminarin and chitosan did not prevent weight loss or reduce the gene expression levels of inflammatory markers, the co-expression patterns between genes within these groups differed to that observed in the basal DSS group. Furthermore, the supplementation with laminarin resulted in increased observed bacterial diversity and increased molar proportions of acetate, with reductions in members of the genus *Escherichia/Shigella*. On its own, laminarin is not sufficient for the prevention of the symptoms and the epithelial damage observed in IBD patients, however, it may have potential as a complementary dietary supplement for the maintenance of remission by preventing the proliferation of potential pathogens and enhancing the volatile fatty acid profile in the colon. A longer-term study is warranted to further assess the anti-inflammatory potential of laminarin following an acute DSS challenge. 

## Figures and Tables

**Table 1:** Effect of the dietary supplement on the pre-challenge performance (least square means with their standard errors).

	Treatment			SEM
	Basal	Laminarin	Chitosan	
Initial BW (kg)	8.93	9.25	9.17	0.169
BW d35 (kg)	21.71	20.64	22.19	0.729
ADG d0-35 (kg/day)	0.360	0.326	0.370	0.016
ADFI d0-35 (kg/day)	0.743 ^a^	0.674 ^b^	0.705 ^ab^	0.020
G:F d0-35 (kg/kg)	0.743	0.674	0.705	0.020

BW, body weight; d, day; ADG, average daily gain; ADFI, average daily feed intake; G:F, gain to feed ratio; FS, faecal score. ^a,b^ Mean values within a row with unlike superscript letters were significantly different (*p* < 0.05).

**Table 2:** The effect of the treatment on pig growth performance and faecal consistency during the DSS challenge period d39–44 (Least square mean with their standard errors).

	Treatment				SEM	Contrasts		
	Control	Basal DSS	Laminarin DSS	Chitosan DSS		1	2	3
Final BW (kg)	26.35	24.47	24.76	24.77	0.382	**	NS	NS
ADG (kg/day)	0.499	0.289	0.321	0.324	0.042	**	NS	NS
ADFI (kg/day)	1.42	1.16	1.02	1.17	0.059	**	NS	NS
G:F (kg/kg)	0.356	0.233	0.317	0.278	0.034	*	NS	NS
FS	2.14	2.43	2.76	2.83	0.066	**	***	***

BW, body weight; ADG, average daily gain; ADFI, average daily feed intake; G:F, gain to feed ratio; FS, faecal score; Contrasts: (1) Control vs. Basal DSS; (2) Basal DSS vs. Laminarin DSS; (3) Basal DSS vs. Chitosan DSS; NS, non-significant; * *p* < 0.05; ** *p* < 0.01; *** *p* < 0.001.

**Table 3:** The effect of the treatment on the pathology score and the goblet and epithelial cell % in the colonic tissue (least square means with their standard errors).

	Treatment				SEM	Contrasts		
	Control	Basal DSS	Laminarin DSS	Chitosan DSS		1	2	3
Pathology score	1.0	2.0	1.7	1.9	0.330	*	NS	NS
Goblet cell %	14.9	27.02	23.12	22.54	2.72	**	NS	NS
Epithelial cell %	85.1	72.99	76.88	77.46	2.72	**	NS	NS

The pathology scores range from 1–5 as follows: 1 = normal, non-ulcerating epithelium with a reduced lamina proprial cell infiltration; 2 = normal with a lamina proprial cell infiltration within the normal spectrum; 3 = colonic samples with focal (single) regions of ulceration; 4 = samples with multiple foci of ulceration interspersed between regions of normal colon and 5 = samples with diffuse ulceration. Contrasts: (1) Control vs. Basal DSS; (2) Basal DSS vs. Laminarin DSS; (3) Basal DSS vs. Chitosan DSS; NS, non-significant; * *p* < 0.05; ** *p* < 0.01.

**Table 4:** The effects of the treatment on gene expression in the colon (least square means with their standard errors).

	Treatment				SEM	Contrasts		
	Control	Basal DSS	Laminarin DSS	Chitosan DSS		1	2	3
*MMP1*	0.245	2.142	0.857	2.371	0.646	*	NS	NS
*IL13*	0.634	1.033	0.916	0.870	0.103	*	NS	NS
*IL23A*	0.487	0.975	1.052	0.740	0.153	*	NS	NS
*MMP2*	0.476	1.113	0.945	1.079	0.268	NS	NS	NS
*MMP9*	0.525	1.065	0.758	1.164	0.256	NS	NS	NS
*CLEC7A*	0.929	1.453	0.943	1.236	0.330	NS	NS	NS
*TLR4*	0.904	1.046	1.554	1.359	0.350	NS	NS	NS
*TJP1*	0.895	0.930	0.867	0.924	0.056	NS	NS	NS
*CDH1*	0.891	1.122	1.017	1.022	0.112	NS	NS	NS
*MARVELD2*	1.480	1.566	1.688	1.765	0.451	NS	NS	NS
*IL10*	0.587	0.906	1.019	0.962	0.159	NS	NS	NS
*IL17A*	0.808	0.747	1.157	1.217	0.288	NS	NS	NS
*IL6*	0.310	1.725	1.356	1.269	0.549	NS	NS	NS
*IL1B*	0.428	0.905	1.170	1.451	0.322	NS	NS	NS
*IFNG*	1.457	0.899	1.081	1.243	0.425	NS	NS	NS
*TGFB1*	0.925	0.870	0.913	0.944	0.156	NS	NS	NS
*MUC4*	1.393	0.780	0.931	1.337	0.247	NS	NS	NS

*MMP1*, matrix metalloproteinase 1; *IL13*, interleukin 13; *IL23A*, interleukin 23 subunit alpha; *MMP2*, matrix metalloproteinase 2; *MMP9*, matrix metalloproteinase 9; *CLEC7A*, C-type lectin domain containing 7A; *TLR4*, Toll-like receptor 4; *TJP1*, tight junction protein 1; *CDH1*, cadherin 1; MARVELD2, MARVEL domain containing 2; *IL10*, interleukin 10; *IL17A*, interleukin 17A; *IL6*, interleukin 6; IL1B, interleukin 1B; *IFNG*, interferon gamma; *TGFB1*, transforming growth factor beta 1; *MUC4*, mucin 4; Contrasts: (1) Control vs. Basal DSS; (2) Basal DSS vs. Laminarin DSS; (3) Basal DSS vs. Chitosan DSS; NS, non-significant; * *p* < 0.05.

**Table 5:** The effect of the treatment on the total volatile fatty acids (VFA) concentrations and molar proportions of VFA in the colonic digesta (least square means with their standard errors).

	Treatment				SEM	Contrasts		
	Control	Basal DSS	Laminarin DSS	Chitosan DSS		1	2	3
Total VFA(mmol/g digesta)	152.58	169.00	158.29	169.83	6.910	NS	NS	NS
Molar proportions								
Acetate	0.64	0.57	0.62	0.58	0.014	**	*	NS
Propionate	0.185	0.218	0.196	0.222	0.010	*	NS	NS
Isobutyrate	0.012	0.013	0.012	0.011	0.002	NS	NS	NS
Butyrate	0.111	0.123	0.110	0.124	0.006	NS	NS	NS
Isovalerate	0.012	0.014	0.012	0.012	0.002	NS	NS	NS
Valerate	0.039	0.061	0.051	0.054	0.006	**	NS	NS
Branched	0.063	0.088	0.075	0.077	0.007	**	NS	NS

Contrasts: (1) Control vs. Basal DSS; (2) Basal DSS vs. Laminarin DSS; (3) Basal DSS vs. Chitosan DSS; NS, non-significant; * *p* < 0.05; ** *p* < 0.01.

**Table 6:** Effect of the treatment on the measures of alpha diversity in the colonic digesta (least square means with their standard errors).

Measure of Diversity	Treatment				SEM	Contrasts ^a^		
	Control	Basal DSS	Laminarin DSS	Chitosan DSS		1	2	3
Observed	195.80	128.17	163.75	160.80	7.13	*	*	NS
Chao1	288.56	196.58	229.36	229.06	9.53	*	NS	NS
ACE	290.97	204.62	236.14	238.28	8.93	*	NS	NS
Shannon	3.74	3.12	3.42	3.31	0.09	*	NS	NS
Simpson	0.93	0.86	0.91	0.89	0.01	*	NS	NS
InvSimpson	18.78	12.91	15.34	12.23	1.52	NS	NS	NS
Fisher	51.93	33.54	41.63	41.44	2.05	**	NS	NS

ACE, abundance-based coverage estimators; ^a^ adjusted *p*-values; Contrasts: (1) Control vs. Basal DSS; (2) Basal DSS vs. Laminarin DSS; (3) Basal DSS vs. Chitosan DSS; NS, non-significant; * *p* < 0.05; ** *p* < 0.01.

**Table 7:** Differentially abundant OTUs at the phylum, family, and the genus levels (least square means with their standard errors).

Taxa	OTU	Treatment				SEM	Contrasts ^a^		
		Control	Basal DSS	Laminarin DSS	Chitosan DSS		1	2	3
P-Deferribacteres	AF059190.1.1451	0.07	0.78	0.27	0.90	0.120	***	NS	NS
P-Cyanobacteria	EU474510.1.1379	0.68	0	0.01	0.02	0.097	***	NS	NS
*F-Lachnospiraceae*	New.CleanUp.ReferenceOTU8	3.722	1.281	1.830	1.476	0.239	*	NS	NS
*F-GR-WP33-58*	AF371949.1.1454	0.851	0.000	0.000	0.014	0.094	***	NS	NS
*F-Synergistaceae*	JQ606918.1.1410	0.003	0.117	1.357	0.163	0.225	*	NS	NS
*F-Uncultured bacterium*	DQ805702.1.1378	0.071	0.000	0.018	0.004	0.009	***	NS	NS
*F-Clostridiales vadin BB60 group*	HQ780759.1.1426	0.416	0.466	1.186	1.465	0.174	NS	NS	*
*G-Phascolarctobacterium*	GQ358246.1.1466	1.011	0.000	0.178	0.097	0.115	**	NS	NS
*G-[Eubacterium] ruminantium group*	EU775472.1.1384	0.514	0.039	0.138	0.012	0.072	**	NS	NS
*G-Oribacterium*	EU461618.1.1389	0.283	0.004	0.000	0.012	0.025	**	NS	NS
*G-Roseburia*	EU466950.1.1385	1.324	0.115	0.179	0.243	0.097	***	NS	NS
*G-Anaerovibrio*	New.CleanUp.ReferenceOTU1	4.293	0.232	1.390	1.056	0.439	***	NS	NS
*G-Bacteroides*	KF842513.1.1417	0.000	0.413	0.455	0.210	0.081	***	NS	NS
*G-Rikenellaceae RC9 gut group*	New.CleanUp.ReferenceOTU14	1.406	0.238	0.585	0.268	0.165	*	NS	NS
*G-Prevotella 1*	GQ448219.1.1401	0.270	0.008	0.007	0.000	0.039	*	NS	NS
*G-Prevotella 7*	EU458732.1.1352	0.444	0.004	0.000	0.000	0.048	*	NS	NS
G-Uncultured bacterium	EF445225.1.1490	0.382	0.012	0.045	0.057	0.047	**	NS	NS
*G-Thalassospira*	New.CleanUp.ReferenceOTU23	0.463	0.000	0.009	0.093	0.047	**	NS	NS
*G-Escherichia-shigella*	KF842027.1.1375	0.538	2.626	0.405	0.594	0.362	**	**	NS
*G-Campylobacter*	AF550648.1.1562	0.019	0.283	0.054	0.104	0.050	**	NS	NS
*G-Mucispirillum*	AF059190.1.1451	0.074	0.799	0.273	0.913	0.122	***	NS	NS
G-Uncultured bacteria	HQ780759.1.1426	0.407	0.474	1.206	1.458	0.176	NS	*	*
G-Uncultured bacterium	JQ184479.1.1344	0.542	0.004	0.008	0.022	0.067	***	NS	NS
*G-Succinivibrio*	KF843489.1.1384	3.745	1.117	9.575	1.261	1.078	NS	*	NS
*G-Succinivibrionaceae UCG-001*	HQ400282.1.1506	13.036	28.345	0.062	13.760	2.736	NS	***	NS
*G-Olsenella*	JQ188553.1.1334	0.099	0.174	0.000	0.012	0.029	NS	*	NS

P, phylum; F, family; G, genus; OTU, operational taxonomic unit; ^a^ adjusted *p*-values; Contrasts: (1) Control vs. Basal DSS; (2) Basal DSS vs. Laminarin DSS; (3) Basal DSS vs. Chitosan DSS; NS, non-significant; * *p* < 0.05, ** *p* < 0.01, *** *p* < 0.001.

**Table 8:** Ingredient and chemical composition of the basal diet ^a^.

Ingredient	g/kg
Whey powder	50.0
Wheat	380.0
Barley	234.0
Soya bean meal	170.0
Full-fat soya bean	120.0
Soya oil	10.0
Vitamins and minerals ^b^	3.0
Salt	3.0
Dicalcium phosphate	13.0
Limestone	11.0
Lysine HCL	4.0
DL-methionine	1.5
L-threonine	1.5
Chemical analysis	
Dry matter	866.1
Crude protein (N × 6.25)	210.6
Ash	48.4
Neutral detergent fibre	115.1
Digestible energy (MJ/kg) *	14.5
Lysine *	14.5
Methionine and cysteine *	8.4
Threonine *	9.1
Tryptophan *	2.5
Calcium *	9.5
Phosphorous *	6.1

HCL, hydrochloride; ^a^ Treatments: (1) basal diet; (2) basal diet DSS, (3) basal diet + 200 ppm laminarin DSS, (4) basal diet + 300 ppm chitosan DSS; ^b^ Provided (mg/kg complete diet): Cu, 100; Fe, 140; Mn, 47; Zn, 120; I, 0·6; Se, 0·3; retinol, 1·8; cholecalciferol, 0·025; α-tocopherol, 67; phytylmenaquinone, 4; cyanocobalamin, 0·01; riboflavin, 2; nicotinic acid, 12; pantothenic acid, 10; choline chloride, 250; thiamin, 2; pyridoxine, 0·015. Celite included at 300 mg/kg complete diet; * Calculated for tabulated nutritional composition [71].

**Table 9:** Porcine oligonucleotide primers used for real-time PCR.

Genes	Accession No.	Forward Primer (5’-3’) Reverse Primer (5’-3’)	Tm
**Cytokines**			
*IL23A*	NM_001130236.1	F:AGGGACTCAGGGACAACAGTC	61.1
		R:GCGAAGGATCTTGAGGCGGAGAAGGAG	68.42
*IL13*	NM_213803.1	F:CCTGGAATCCCTCATCAACAT	57.41
		R:AGGGCGCTCAGGATCCT	59.66
*IL10*	NM_214041.1	F:GCCTTCGGCCCAGTGAA	57.6
		R:AGAGACCCGGTCAGCAACAA	59.4
*IL6*	NM_214399.1	F:GACAAAGCCACCACCCCTAA	59.8
		R:CTCGTTCTGTGACTGCAGCTTATC	62.7
*IFNG*	NM_213948.1	F:TCTAACCTAAGAAAGCGGAAGAGA	61.1
		R:TTGCAGGCATGACAATTA	61.5
*IL1B*	NM_214055.1	F:TTGAATTCGAGTCTGCCCTGT	59.65
		R:CCCAGGAAGACGGGCTTT	59.24
*TGFB1*	NM_214015.1	F:AGGGCTACCATGCCAATTTCT	59.71
		R:CGGGTTGTGCTGGTTGTACA	60.82
*IL17A*	NM_001005729.1	F:CCCTGTCACTGCTGCTTCTG	60.95
		R:TCATGATTCCCGCCTTCAC	57.52
**Matrix metalloproteinase’s**			
*MMP2*	NM_214192.2	F:CTTCAAGGGCGCGTATTACC	59.06
		R:GCCAGTCGGATTTGATGCTT	58.9
*MMP9*	NM_001038004.1	F:GGCTACAGCCTGTTCCTTGTG	61.22
		R:GGCACGGTTGAGTGATCTAAGC	61.56
*MMP1*	NM_001166229.1	F:GGACCGTGCCATTGAGAA	57.29
		R:CCTCGGAGACCTTGGTGAAC	60.04
**Tight junctions**			
*TJP1*	XM_005659811.1	F:TGAGAGCCAACCATGTCTTGAA	59.9
		R:CTCAGACCCTCTCTGTCT	60.0
*CDH1*	NM_001163060.1	F:GCCTGGCAACTGAGCTGACT	62.74
		R:CCTCCCTCCTTCAGAATTTTC	55.93
*MARVELD2*	NM_001243948.1	F: CAGGCACCACGACGAAGTC	61.02
		R: AAGCGTCTGGAAGGTTCTTACG	60.61
*Mucin*			
*MUC4*	XM_001926442.1	F:GATGCCCTGGCCACAGAA	63.3
		R:TGATTCAAGGTAGCATTCATTTGC	62.4
**Pattern recognition receptors**			
*TLR4*	NM_001293317.1	F:TGCATGGAGCTGAATTTCTACAA	58.6
		R:GATAATCCAGCACCTGCAGTTC	59.9
*CLEC7A*	NM_001145866.1	F:GCACATCATTAGCTTCCTGGAA	58.72
		R:GGAGCTGTCTATCTTCAGGAGA	58.44
**Reference genes**			
*PPIA*	NM_214353.1	F:CGGTCCTGGCATCTTGT	59.67
		R:TGGCAGTGCAAATGAAAAACTG	59.61
*ACTB*	AY550069.1	F:CAAATGCTTCTAGGCGGCGGACTGT	60.9
		R:TCTCATTTTCTGCGCAAGTTAGG	59.5

IL23A, interleukin 23 subunit alpha; IL13, interleukin 13; IL10, interleukin 10; IL6, interleukin 6; IFNG, interferon gamma; IL1B, interleukin 1B; TGFB1, transforming growth factor beta 1; IL17A, interleukin 17A; MMP2, matrix metalloproteinase 2; MMP9, matrix metalloproteinase 9; MMP1, matrix metalloproteinase 1; TJP1, tight junction protein 1; CDH1, cadherin 1; MUC4, mucin 4; TLR4, Toll-like receptor 4; CLEC7A, c-type lectin domain containing 7A; PPIA, peptidylprolyl isomerase; ACTB, β-actin.

**Table 10:** The forward primer adaptor sequence and the six base pair tags/unique barcode sequences.

Primer ID	Forward Primer 5’-3’	Unique 6 Nucleotide Tag
PA-V4-1	CCATCTCATCCCTGCGTGTCTCCGACTCAG	AGCAGC
PA-V4-2	CCATCTCATCCCTGCGTGTCTCCGACTCAG	CTCAGC
PA-V4-3	CCATCTCATCCCTGCGTGTCTCCGACTCAG	AGAGAG
PA-V4-4	CCATCTCATCCCTGCGTGTCTCCGACTCAG	AGATGC
PA-V4-5	CCATCTCATCCCTGCGTGTCTCCGACTCAG	AGCATG
PA-V4-6	CCATCTCATCCCTGCGTGTCTCCGACTCAG	ATCATC
PA-V4-7	CCATCTCATCCCTGCGTGTCTCCGACTCAG	ATCTGC
PA-V4-8	CCATCTCATCCCTGCGTGTCTCCGACTCAG	ATGAGC
PA-V4-9	CCATCTCATCCCTGCGTGTCTCCGACTCAG	ATGATG
PA-V4-10	CCATCTCATCCCTGCGTGTCTCCGACTCAG	ATGCAG
PA-V4-11	CCATCTCATCCCTGCGTGTCTCCGACTCAG	ATGCTC
PA-V4-12	CCATCTCATCCCTGCGTGTCTCCGACTCAG	CAGAGC
PA-V4-13	CCATCTCATCCCTGCGTGTCTCCGACTCAG	CAGATG
PA-V4-14	CCATCTCATCCCTGCGTGTCTCCGACTCAG	CAGCAG
PA-V4-15	CCATCTCATCCCTGCGTGTCTCCGACTCAG	CAGCTC
PA-V4-16	CCATCTCATCCCTGCGTGTCTCCGACTCAG	CATCTG
PA-V4-17	CCATCTCATCCCTGCGTGTCTCCGACTCAG	CATGAG
PA-V4-18	CCATCTCATCCCTGCGTGTCTCCGACTCAG	CTCATG
PA-V4-19	CCATCTCATCCCTGCGTGTCTCCGACTCAG	CTGATC
PA-V4-20	CCATCTCATCCCTGCGTGTCTCCGACTCAG	CTGCTG

## Supplementary Materials

The following are available online at https://www.mdpi.com/1660-3397/18/5/262/s1, Table S1: title, Video S1: Effect of treatment on the relative abundance of bacterial taxa at phylum, family, and genus levels.
